# Depression and Inflammatory Periodontal Disease Considerations—An Interdisciplinary Approach

**DOI:** 10.3389/fpsyg.2016.00347

**Published:** 2016-03-23

**Authors:** Alexandrina L. Dumitrescu

**Affiliations:** Private Dental PracticeBucharest, Romania

**Keywords:** depression, periodontitis, inflammation, cytokines, periodontal disease, smoking, oxidative stress, psychology

## Introduction

Periodontal disease, a bacterially mediated inflammatory disease of the gingival and adjacent periodontal attachment apparatus, represents, after dental caries, the leading cause of tooth loss among adults in developed countries due to the destruction of the periodontal ligament and the loss of the adjacent supporting bone, the tissues which support the teeth (Pihlstrom et al., 2005).

Depressive disorders, the most commonly diagnosed conditions in psychiatry (Ustün et al., 2004; Kessler and Bromet, 2013), include, according to the fifth edition of the Diagnostic and Statistical Manual of Mental Disorders (DSM-5): disruptive mood dysregulation disorder, major depressive disorder (including major depressive episode) an extensive prevalent disorder ranked third among the primary causes of global illness (Mathers and Loncar, 2006), persistent depressive disorder (dysthymia), premenstrual dysphoric disorder, substance/medication-induced depressive disorder, depressive disorder due to another medical condition, other specified depressive disorder, and unspecified depressive disorder (American Psychiatric Association, 2013; Patten, 2013).

**Figure 1:**
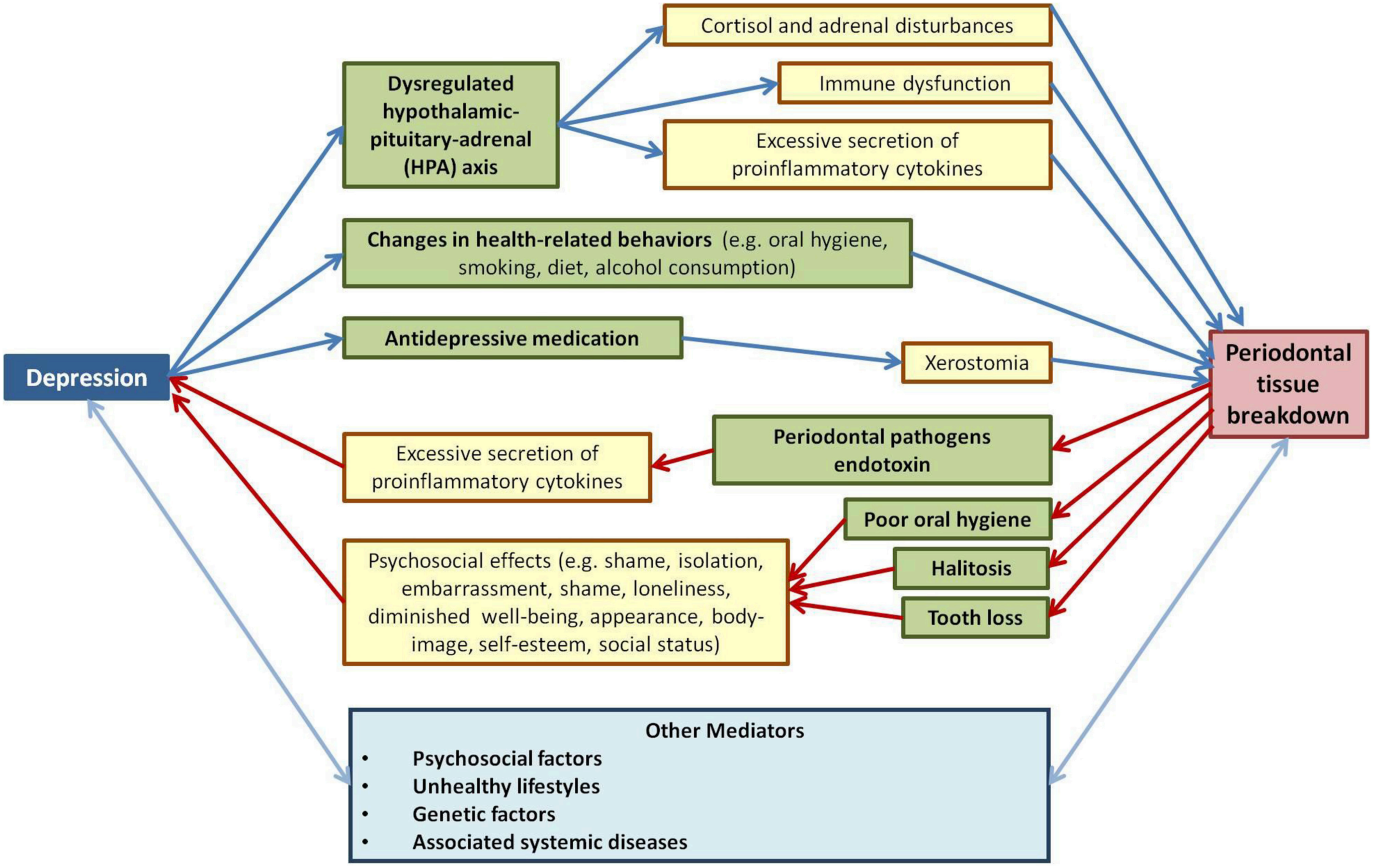
**Review of reported results related to the biological and psychosocial mechanisms underlying the depression-periodontal disease bidirectional connection**.

The aim of this article is to summarize the current knowledge about the periodontal disease—depression relationship and to discuss the plausible mechanisms underlying this possible bidirectional association, by which each disease may contribute to the other (Figure 1).

## The periodontal disease-depression association's studies

An extensive body of clinical research (Monteiro da Silva et al., 1996; Moss et al., 1996; Genco et al., 1999; Ronderos and Ryder, 2004; Dosumu et al., 2005; Klages et al., 2005; Saletu et al., 2005; Johannsen et al., 2006, 2007; Rosania et al., 2009; Ababneh et al., 2010; Li et al., 2011; López et al., 2012) and experimental animal models (Breivik et al., 2006) documents the causal relationships between periodontitis and depression. Moreover, it has been revealed that clinical depression may also have a negative effect on periodontal treatment outcomes (Elter et al., 2002), paralleling other research indicating that psychosocial factors are predictive not only of surgical outcome, but also play a significant role in postoperative recovery (Rosenberger et al., 2006). Furthermore, antidepressants, such as fluoxetine, a selective serotonin reuptake inhibitor, have demonstrated suppressive effects on the inflammatory response and on periodontal disease severity not only in a rat ligature-induced periodontitis model (Branco-de-Almeida et al., 2012; Aguiar et al., 2013; Galli et al., 2013), but also in patients with periodontitis with clinical depression (Bhatia et al., 2015). In contrast, several clinical studies (Anttila et al., 2001; Persson et al., 2003; Solis et al., 2004, 2014; Castro et al., 2006; Cakmak et al., 2014) and experimental animal model studies (Soletti et al., 2009) failed to demonstrate the periodontal disease—depression connection, possibly because of the lack of consideration for relevant common mediators.

## Relevant mediators of the periodontal disease-depression association

Periodontal disease and depression are sharing common risk factors within the context of the wider socio-environmental milieu and adopting a collaborative approach (e.g., the common risk factor approach) is more rational than one that is disease specific (Sheiham and Watt, 2000; Watt, 2007; Petersen and Ogawa, 2012; Thomson et al., 2012; Watt and Petersen, 2012; Watt and Sheiham, 2012; Bentley et al., 2014).

The prevalence and severity of both periodontitis and depression, are associated with several social determinants such as older age (Holtfreter et al., 2009; Genco and Borgnakke, 2013; Allan et al., 2014; Kassebaum et al., 2014a), low socioeconomic status (Haustein, 2005; Borrell and Crawford, 2012; Haas et al., 2012; Thomson et al., 2012), low educational level (Kocher et al., 2005; Boillot et al., 2011; Eke et al., 2012; Hong and Tian, 2014), and ethnicity (Dunlop et al., 2003; Eke et al., 2012).

Unhealthy lifestyles, such as smoking and alcohol consumption have been shown to be risk factors for periodontal disease (Pitiphat et al., 2003; Tezal et al., 2004; Chambrone et al., 2013; Genco and Borgnakke, 2013; Fiorini et al., 2014) and also for depression (Paperwalla et al., 2004; Luger et al., 2014; Klimkiewicz et al., 2015). Moreover, scientific reports have shown that poor diet and a lack of exercise contribute to the genesis and course of depression (Jacka and Berk, 2012) and are associated with a higher periodontitis prevalence (Nishida et al., 2000; Moynihan and Petersen, 2004; Al-Zahrani et al., 2005). Furthermore, animal models and clinical studies have highlighted causal relationships between sleep deprivation and severity of periodontitis on the one hand (Grover et al., 2015; Nakada et al., 2015) and between sleep deprivation and mood changes on the other hand (Costae Silva, 2006; Turek, 2007; Kronfeld-Schor and Einat, 2012). Moreover, stress, distress, and psychological resistance (personality, coping and social support) were connected with periodontal disease (Genco et al., 1999; Dumitrescu, 2006; Peruzzo et al., 2007; Warren et al., 2014) and depression (Hammen, 2005; Klein et al., 2011; Rosenquist et al., 2011; Luca et al., 2013).

Common genetic contributing factors have been also identified for the two diseases. Involvement of genetic polymorphism of brain-derived neurotropic factor (*BDNF*) and serotonin (5-hydroxytryptamine *5-HT*) has been reported in depression studies (Roy et al., 2014). In the same time, BDNF genotype GG was correlated with higher levels of BDNF, TNF-α, and the chemokine CXCL10 in patients with chronic periodontitis (Corrêa et al., 2014), while 5-HTTLPR polymorphism was associated with aggressive periodontitis (Costa et al., 2008; Mendes et al., 2013). A recent large sized *in silico* data analysis performed by Kao et al. (2011) has prioritized 169 genes out of 5055 candidate genes for depression. Besides BDNF and 5-HTTLPR, among top prioritized gene products related also to alveolar bone resorption and periodontal involvement being Tumour necrosis factor (TNF) polymorphism (Khosravi et al., 2013; Ding et al., 2014).

An examination of the research investigating the relationships between oral health and general health (Petersen, 2006; Kandelman et al., 2008) revealed a strong relationship between periodontal health or disease and various medical conditions (e.g., metabolic syndrome, cardiovascular disease, adverse pregnancy outcomes, respiratory disease, rheumatoid arthritis, cancer, inflammatory bowel disease, and Alzheimer disease; Williams and Offenbacher, 2000; Aarabi et al., 2015; Bascones-Martínez et al., 2015; Hatipoglu et al., 2015; Leech and Bartold, 2015; Nagpal et al., 2015; Payne et al., 2015; Javed and Warnakulasuriya, 2016). In the same time, an emerging body of evidence demonstrates a co-morbidity of depression with severe physical disorders with high mortality rates, such as cancer, stroke, and acute coronary syndrome (Kang et al., 2015) and particularly among patients with multiple physical disorders (Sobel et al., 2005; Maes et al., 2011a; Smith et al., 2014; Wu et al., 2014; Kang et al., 2015). Among them, several systemic medical conditions [Human immunodeficiency (HIV) infection, diabetes mellitus, obesity] are considered well documented risk factors for periodontal disease (Grossi et al., 1994; Ritchie, 2007; Kinane et al., 2008; Genco and Borgnakke, 2013) and depression (Pratt and Brody, 2014; Semenkovich et al., 2015; Serafini et al., 2015).

## Depression as a cause of periodontal disease

Several mechanisms have been proposed to explain the mechanism by which depression plays a causal role in the in the $aetiology of inflammatory periodontal disease:

- Depression supports a chronic **dysregulated hypothalamic-pituitary-adrenal (HPA) axis** and further determines cortisol and adrenal disturbances, as well as immune dysfunction and excessive secretion of proinflammatory cytokines (Heim et al., 2008; Miller et al., 2009; Guerry and Hastings, 2011; Belvederi Murri et al., 2014; Moylan et al., 2014). Through these processes, depression might affect progression of periodontal infections in patients susceptible to periodontitis (Saletu et al., 2005) and might be associated with a worse treatment outcome through a delay of wound healing (Bosch et al., 2007). Moreover, animal studies have demonstrated that various classes of antidepressants can reduce levels of oxidative stress markers (Eren et al., 2007a,b; Maes et al., 2011a; Leonard and Maes, 2012), increase several endogenous antioxidants (Maes et al., 2011a) and also decrease the periodontal disease severity (Branco-de-Almeida et al., 2012; Aguiar et al., 2013; Galli et al., 2013). Captivatingly, these biological processes have been revealed to participate to the aetiology of depression and periodontal disease co-morbidities, as well, and thus may represent a bridge between these pathologies (Maes et al., 2011b; Bullon et al., 2014; Rossetti et al., 2014; Jani et al., 2015; Kang et al., 2015).
- **Changes in health-related behaviors**, such as oral hygiene, smoking, diet, alcohol consumption that occurs in depressed patients can also be related to the occurrence of periodontal disease (Kurer et al., 1995; D'Alessandro et al., 2014; Peltzer and Pengpid, 2014; Yuen et al., 2014; Alkan et al., 2015). However, one study failed to find a relationship between depression and dental plaque levels (Marques-Vidal and Milagre, 2006).
- Moreover, the **antidepressive medication may also lead to xerostomia** (Friedlander and Norman, 2002; Thomson et al., 2006; Macedo et al., 2014), alterations in gingival circulation and changes in saliva composition that might result in an exacerbation of periodontitis. However, further research is required in this area as some studies have found a causal relationship between reduced salivary flow and periodontal disease (Farsi et al., 2008; Márton et al., 2008; Samnieng et al., 2012), whereas not in others (Hirotomi et al., 2006; Syrjälä et al., 2011).

## Periodontal disease as a cause of depression

Finally, periodontal disease may contribute to the onset of depression through different pathways:

- Depression is associated with a chronic, low-grade inflammatory response, activation of cell-mediated immunity, and compensatory anti-inflammatory reflex system, as well as an augmentation of oxidative and nitrosative stress, which contribute to neuroprogression in the disorder (Berk et al., 2013; Slavich and Irwin, 2014). Recent meta-analyses revealed that depressive patients have higher serum levels of pro-inflammatory cytokines such interleukin (IL)-1, IL-6, and tumor necrosis factor alpha (TNFα; Howren et al., 2009; Dowlati et al., 2010; Maes, 2011; Hiles et al., 2012; Valkanova et al., 2013; Sarkar and Schaefer, 2014; Black and Miller, 2015) as wells increased levels of acute phase proteins (e.g., C-reactive protein, complement factors, chemokines; Berk et al., 1997; Pasco et al., 2010; Cekici et al., 2014). Moreover, the administration of pro-inflammatory cytokines and lipopolysaccharide has been able to induce depressive-like behaviors in rodent studies (Manosso et al., 2013; Mello et al., 2013; Kurosawa et al., 2015; Zhu et al., 2015). Critically, periodontal disease is also associated with high levels of systemic inflammation, in particularly, interleukin-6 (IL-6), TNF-α, and C-reactive protein (CRP; Pussinen et al., 2007; Bansal et al., 2014) that may potentiate inflammatory and oxidative and nitrosative stress processes and thus may lead to a vulnerability to depression (Battino et al., 1999; Chapple and Matthews, 2007; Berk et al., 2013; Bullon et al., 2014).
- Furthermore, periodontal disease may increase the risk for depression through the psychosocial effects (e.g., shame, isolation, embarrassment, loneliness) of poor oral hygiene and halitosis, frequent characteristics of patients with periodontal disease (Morita and Wang, 2001; Tsai et al., 2008; Pham et al., 2012; Silveira et al., 2012; Durham et al., 2013; Guentsch et al., 2014).
- Periodontal disease is also one of the leading causes of edentulousness due to the inflammatory destruction of the tooth supporting tissues: the periodontal ligament and the alveolar bone (Kassebaum et al., 2014b). As the contour and aesthetics of the face are maintained by natural teeth and alveolar bone, tooth loss may affect the patients' quality of life, not only due to dental loss of chewing functionality, but also when it impairs their body-image, diminishes self-esteem, and social status (Gerritsen et al., 2010; Saintrain and de Souza, 2012; Al-Harthi et al., 2013). This is supported by the positive correlations between tooth loss and depression that have been revealed by a high number of studies (Anttila et al., 2001; Davis et al., 2001; Persson et al., 2003; Rosania et al., 2009; Coles et al., 2011; Matthews et al., 2011; Okoro et al., 2012; Urzua et al., 2012; Yamamoto et al., 2012; Luo et al., 2015; Roohafza et al., 2015), psychological counseling being necessary to be provided along with periodontal and prosthetic treatment (Priyadarshini et al., 2014).

## Conclusion

An interdisciplinary approach in psychoimmunology and periodontology has been used to highlight the biological and psychosocial mechanism and mediators of the depression and periodontitis connection, in order to call attention to potential new therapeutic strategies for both depressed individuals and periodontal disease patients.

## Author contributions

The author confirms being the sole contributor of this work and approved it for publication.

### Conflict of interest statement

The author declares that the research was conducted in the absence of any commercial or financial relationships that could be construed as a potential conflict of interest. The reviewer PV and handling Editor declared a current collaboration and the handling Editor states that the process nevertheless met the standards of a fair and objective review.

## References

[B1] Aarabi G. Eberhard J. Reissmann D. R. Heydecke G. Seedorf U. (2015). Interaction between periodontal disease and atherosclerotic vascular disease - Fact or fiction? Atherosclerosis 241, 555–560. 10.1016/j.atherosclerosis.2015.04.819 26100678

[B2] Ababneh K. T. Taha A. H. Abbadi M. S. Karasneh J. A. Khader Y. S. (2010). The association of aggressive and chronic periodontitis with systemic manifestations and dental anomalies in a jordanian population: a case control study. Head Face Med. 6:30. 10.1186/1746-160X-6-30 21190556 PMC3022550

[B3] Aguiar J. C. Gomes E. P. Fonseca-Silva T. Velloso N. A. Vieira L. T. Fernandes M. F.. (2013). Fluoxetine reduces periodontal disease progression in a conditioned fear stress model in rats. J. Periodontal Res. 48, 632–637. 10.1111/jre.12049 23425324

[B4] Al-Harthi L. S. Cullinan M. P. Leichter J. W. Thomson W. M. (2013). The impact of periodontitis on oral health-related quality of life: a review of the evidence from observational studies. Aust. Dent. J. 58, 274–277. 10.1111/adj.12076 23981206

[B5] Alkan A. Cakmak O. Yilmaz S. Cebi T. Gurgan C. (2015). Relationship between psychological factors and oral health status and behaviours. Oral Health Prev. Dent. 13, 331–339. 10.3290/j.ohpd.a32679. 25197739

[B6] Allan C. E. Valkanova V. Ebmeier K. P. (2014). Depression in older people is underdiagnosed. Practitioner 258, 19–22. 25065018

[B7] Al-Zahrani M. S. Borawski E. A. Bissada N. F. (2005). Periodontitis and three health-enhancing behaviors: maintaining normal weight, engaging in recommended level of exercise, and consuming a high-quality diet. J. Periodontol. 76, 1362–1366. 10.1902/jop.2005.76.8.1362 16101370

[B8] American Psychiatric Association (2013). Diagnostic and Statistical Manual of Mental Disorders, 5th Edn. Washington, DC: American Psychiatric Association.

[B9] Anttila S. S. Knuuttila M. L. Sakki T. K. (2001). Relationship of depressive symptoms to edentulousness, dental health, and dental health behavior. Acta Odontol. Scand. 59, 406–412. 10.1080/000163501317153275 11831492

[B10] Bansal T. Pandey A. Deepa D. Asthana A. K. (2014). C-Reactive Protein (CRP) and its association with periodontal disease: a brief review. J. Clin. Diagn. Res. 8, ZE21–ZE24. 10.7860/JCDR/2014/8355.4646 25177663 PMC4149169

[B11] Bascones-Martínez A. Muñoz-Corcuera M. Bascones-Ilundain J. (2015). [Diabetes and periodontitis: a bidirectional relationship]. Med. Clin. (Barc) 145, 31–35. 10.1016/j.medcli.2014.07.019 25192582

[B12] Battino M. Bullon P. Wilson M. Newman H. (1999). Oxidative injury and inflammatory periodontal diseases: the challenge of anti-oxidants to free radicals and reactive oxygen species. Crit. Rev. Oral Biol. Med. 10, 458–476. 10.1177/10454411990100040301 10634583

[B13] Belvederi Murri M. Pariante C. Mondelli V. Masotti M. Atti A. R. Mellacqua Z.. (2014). HPA axis and aging in depression: systematic review and meta-analysis. Psychoneuroendocrinology 41, 46–62. 10.1016/j.psyneuen.2013.12.004 24495607

[B14] Bentley S. M. Pagalilauan G. L. Simpson S. A. (2014). Major depression. Med. Clin. North Am. 98, 981–1005. 10.1016/j.mcna.2014.06.013 25134869

[B15] Berk M. Wadee A. A. Kuschke R. H. O'Neill-Kerr A. (1997). Acute phase proteins in major depression. J. Psychosom. Res. 43, 529–534. 10.1016/S0022-3999(97)00139-6 9394269

[B16] Berk M. Williams L. J. Jacka F. N. O'Neil A. Pasco J. A. Moylan S.. (2013). So depression is an inflammatory disease, but where does the inflammation come from? BMC Med. 11:200. 10.1186/1741-7015-11-200 24228900 PMC3846682

[B17] Bhatia A. Sharma R. K. Tewari S. Khurana H. Narula S. C. (2015). Effect of fluoxetine on periodontal status in patients with depression: a cross-sectional observationa study. J. Periodontol. 86, 927–935. 10.1902/jop.2015.140706 25812910

[B18] Black C. Miller B. J. (2015). Meta-analysis of cytokines and chemokines in suicidality: distinguishing suicidal versus nonsuicidal patients. Biol. Psychiatry 78, 28–37. 10.1016/j.biopsych.2014.10.014 25541493

[B19] Boillot A. El Halabi B. Batty G. D. Rangé H. Czernichow S. Bouchard P. (2011). Education as a predictor of chronic periodontitis: a systematic review with meta-analysis population-based studies. PLoS ONE 6:e21508. 10.1371/journal.pone.0021508 21814546 PMC3140980

[B20] Borrell L. N. Crawford N. D. (2012). Socioeconomic position indicators and periodontitis: examining the evidence. Periodontol. 2000 58, 69–83. 10.1111/j.1600-0757.2011.00416.x 22133367 PMC3233193

[B21] Bosch J. A. Engeland C. G. Cacioppo J. T. Marucha P. T. (2007). Depressive symptoms predict mucosal wound healing. Psychosom. Med. 69, 597–605. 10.1097/PSY.0b013e318148c682 17766687

[B22] Branco-de-Almeida L. S. Franco G. C. Castro M. L. Dos Santos J. G. Anbinder A. L. Cortelli S. C.. (2012). Fluoxetine inhibits inflammatory response and bone loss in a rat model of ligature-induced periodontitis. J. Periodontol. 83, 664–671. 10.1902/jop.2011.110370 21966942 PMC3364595

[B23] Breivik T. Gundersen Y. Myhrer T. Fonnum F. Osmundsen H. Murison R.. (2006). Enhanced susceptibility to periodontitis in an animal model of depression: reversed by chronic treatment with the anti-depressant tianeptine. J. Clin. Periodontol. 33, 469–477. 10.1111/j.1600-051X.2006.00935.x 16820034

[B24] Bullon P. Newman H. N. Battino M. (2014). Obesity, diabetes mellitus, atherosclerosis and chronic periodontitis: a shared pathology via oxidative stress and mitochondrial dysfunction? Periodontol. 2000 64, 139–153. 10.1111/j.1600-0757.2012.00455.x 24320961

[B25] Cakmak O. Alkan B. A. Ozsoy S. Sen A. Abdulrezzak U. (2014). Association of gingival crevicular fluid cortisol/dehydroepiandrosterone levels with periodontal status. J. Periodontol. 85, e287–e294. 10.1902/jop.2014.130787 24669848

[B26] Castro G. D. Oppermann R. V. Haas A. N. Winter R. Alchieri J. C. (2006). Association between psychosocial factors and periodontitis: a case-control study. J. Clin. Periodontol. 33, 109–114. 10.1111/j.1600-051X.2005.00878.x 16441734

[B27] Cekici A. Kantarci A. Hasturk H. Van Dyke T. E. (2014). Inflammatory and immune pathways in the pathogenesis of periodontal disease. Periodontol. 2000 64, 57–80. 10.1111/prd.12002 24320956 PMC4500791

[B28] Chambrone L. Preshaw P. M. Rosa E. F. Heasman P. A. Romito G. A. Pannuti C. M.. (2013). Effects of smoking cessation on the outcomes of non-surgical periodontal therapy: a systematic review and individual patient data meta-analysis. J. Clin. Periodontol. 40, 607–615. 10.1111/jcpe.12106 23590649

[B29] Chapple I. L. Matthews J. B. (2007). The role of reactive oxygen and antioxidant species in periodontal tissue destruction. Periodontol. 2000 43, 160–232. 10.1111/j.1600-0757.2006.00178.x 17214840

[B30] Coles E. Chan K. Collins J. Humphris G. M. Richards D. Williams B.. (2011). Decayed and missing teeth and oral-health-related factors: predicting depression in homeless people. J. Psychosom. Res. 71, 108–112. 10.1016/j.jpsychores.2011.01.004 21767692

[B31] Corrêa J. D. Pereira D. S. Madeira M. F. Queiroz-Junior C. M. Souza D. G. Teixeira M. M.. (2014). Brain-derived neurotrophic factor in chronic periodontitis. Mediators Inflamm. 2014:373765. 10.1155/2014/373765 25587209 PMC4283396

[B32] Costa J. E. Gomes C. C. Cota L. O. Pataro A. L. Silva J. F. Gomez R. S.. (2008). Polymorphism in the promoter region of the gene for 5-HTT in individuals with aggressive periodontitis. J. Oral Sci. 50, 193–198. 10.2334/josnusd.50.193 18587210

[B33] Costae Silva J. A. (2006). Sleep disorders in psychiatry. Metabolism 55, S40–S44. 10.1016/j.metabol.2006.07.012 16979426

[B34] D'Alessandro G. Cremonesi I. Alkhamis N. Piana G. (2014). Correlation between oral health in disabled children and depressive symptoms in their mothers. Eur. J. Paediatr. Dent. 15, 303–308. 10.1037/11877-005 25306149

[B35] Davis D. M. Fiske J. Scott B. Radford D. R. (2001). The emotional effects of tooth loss in a group of partially dentate people: a quantitative study. Eur. J. Prosthodont. Restor. Dent. 9, 53–57. 10.1002/j.1875-595x.2001.tb00860.x 11803889

[B36] Ding C. Ji X. Chen X. Xu Y. Zhong L. (2014). TNF-α gene promoter polymorphisms contribute to periodontitis susceptibility: evidence from 46 studies. J. Clin. Periodontol. 41, 748–759. 10.1111/jcpe.12279 24905365

[B37] Dosumu O. O. Dosumu E. B. Arowojolu M. O. Babalola S. S. (2005). Rehabilitative management offered Nigerian localized and generalized aggressive periodontitis patients. J. Contemp. Dent. Pract. 6, 40–52. 16127471

[B38] Dowlati Y. Herrmann N. Swardfager W. Liu H. Sham L. Reim E. K.. (2010). A meta-analysis of cytokines in major depression. Biol. Psychiatry 67, 446–457. 10.1016/j.biopsych.2009.09.033 20015486

[B39] Dumitrescu A. L. (2006). Psychological perspectives on the pathogenesis of periodontal disease. Rom. J. Intern. Med. 44, 241–260. 18386604

[B40] Dunlop D. D. Song J. Lyons J. S. Manheim L. M. Chang R. W. (2003). Racial/ethnic differences in rates of depression among preretirement adults. Am. J. Public Health. 93, 1945–1952. 10.2105/AJPH.93.11.1945 14600071 PMC1199525

[B41] Durham J. Fraser H. M. McCracken G. I. Stone K. M. John M. T. Preshaw P. M. (2013). Impact of periodontitis on oral health-related quality of life. J. Dent. 41, 370–376. 10.1016/j.jdent.2013.01.008 23357646

[B42] Eke P. I. Dye B. A. Wei L. Thornton-Evans G. O. Genco R. J. CDC Periodontal Disease Surveillance workgroup (2012). Prevalence of periodontitis in adults in the United States: 2009 and 2010. J. Dent. Res. 91, 914–920. 10.1177/0022034512457373 22935673

[B43] Elter J. R. White B. A. Gaynes B. N. Bader J. D. (2002). Relationship of clinical depression to periodontal treatment outcome. J. Periodontol. 73, 441–449. 10.1902/jop.2002.73.4.441 11990446

[B44] Eren I. Naziroǧlu M. Demirdaş A. (2007b). Protective effects of lamotrigine, aripiprazole and escitalopram on depression-induced oxidative stress in rat brain. Neurochem. Res. 32, 1188–1195. 10.1007/s11064-007-9289-x 17401662

[B45] Eren I. Naziroǧlu M. Demirdaş A. Celik O. Uǧuz A. C. Altunbaşak A.. (2007a). Venlafaxine modulates depression-induced oxidative stress in brain and medulla of rat. Neurochem. Res. 32, 497–505. 10.1007/s11064-006-9258-9 17268845

[B46] Farsi N. Al Amoudi N. Farsi J. Bokhary S. Sonbul H. (2008). Periodontal health and its relationship with salivary factors among different age groups in a Saudi population. Oral Health Prev. Dent. 6, 147–154. 10.3290/j.ohpd.a13517 18637392

[B47] Fiorini T. Musskopf M. L. Oppermann R. V. Susin C. (2014). Is there a positive effect of smoking cessation on periodontal health? Syst. Rev. J. Periodontol. 85, 83–91. 10.1902/jop.2013.130047 23600995

[B48] Friedlander A. H. Norman D. C. (2002). Late-life depression: psychopathology, medical interventions., and dental implications. Oral Surg. Oral Med. Oral Pathol. Oral Radiol. Endod. 94, 404–412. 10.1067/moe.2002.122434 12374911

[B49] Galli C. Macaluso G. Passeri G. (2013). Serotonin: a novel bone mass controller have implications for alveolar bone. J. Negat. Results Biomed. 12:12. 10.1186/1477-5751-12-12 23964727 PMC3766083

[B50] Genco R. J. Borgnakke W. S. (2013). Risk factors for periodontal disease. Periodontol. 2000 62, 59–94. 10.1111/j.1600-0757.2012.00457.x 23574464

[B51] Genco R. J. Ho A. W. Grossi S. G. Dunford R. G. Tedesco L. A. (1999). Relationship of stress, distress and inadequate coping behaviors to periodontal disease. J. Periodontol. 70, 711–723. 10.1902/jop.1999.70.7.711 10440631

[B52] Gerritsen A. E. Allen P. F. Witter D. J. Bronkhorst E. M. Creugers N. H. (2010). Tooth loss and oral health-related quality of life: a systematic review and meta-analysis. Health Qual. Life Outcomes 8:126. 10.1186/1477-7525-8-126 21050499 PMC2992503

[B53] Grossi S. G. Zambon J. J. Ho A. W. Koch G. Dunford R. G. Machtei E. E.. (1994). Assessment of risk for periodontal disease. I. Risk indicators for attachment loss. J. Periodontol. 65, 260–267. 10.1902/jop.1994.65.3.260 8164120

[B54] Grover V. Malhotra R. Kaur H. (2015). Exploring association between sleep deprivation and chronic periodontitis: a pilot study. J. Indian Soc. Periodontol. 19, 304–307. 10.4103/0972-124X.154173 26229272 PMC4520116

[B55] Guentsch A. Pfister W. Cachovan G. Raschke G. Kuepper H. Schaefer O.. (2014). Oral prophylaxis and its effects on halitosis-associated and inflammatory parameters in patients with chronic periodontitis. Int. J. Dent. Hyg. 12, 199–207. 10.1111/idh.12063 24314016

[B56] Guerry J. D. Hastings P. D. (2011). In search of HPA axis dysregulation in child and adolescent depression. Clin. Child Fam. Psychol. Rev. 14, 135–160. 10.1007/s10567-011-0084-5 21290178 PMC3095794

[B57] Haas A. N. Gaio E. J. Oppermann R. V. Rösing C. K. Albandar J. M. Susin C. (2012). Pattern and rate of progression of periodontal attachment loss in an urban population of South Brazil: a 5-years population-based prospective study. J. Clin. Periodontol. 39, 1–9. 10.1111/j.1600-051X.2011.01818.x 22093104

[B58] Hammen C. (2005). Stress and depression. Annu. Rev. Clin. Psychol. 1, 293–319. 10.1146/annurev.clinpsy.1.102803.143938 17716090

[B59] Hatipoglu H. Yaylak F. Gungor Y. (2015). A brief review on the periodontal health in metabolic syndrome patients. Diabetes Metab. Syndr. 9, 124–126. 10.1016/j.dsx.2015.02.007 25796974

[B60] Haustein K. O. (2005). [Smoking and low socio-economic status]. Gesundheitswesen 67, 630–637. 10.1055/s-2005-858608 16217717

[B61] Heim C. Newport D. J. Mletzko T. Miller A. H. Nemeroff C. B. (2008). The link between childhood trauma and depression: insights from HPA axis studies in humans. Psychoneuroendocrinology 33, 693–710. 10.1016/j.psyneuen.2008.03.008 18602762

[B62] Hiles S. A. Baker A. L. de Malmanche T. Attia J. (2012). A meta-analysis of differences in IL-6 and IL-10 between people with and without depression: exploring the causes of heterogeneity. Brain Behav. Immun. 26, 1180–1188. 10.1016/j.bbi.2012.06.001 22687336

[B63] Hirotomi T. Yoshihara A. Ogawa H. Ito K. Igarashi A. Miyazaki H. (2006). A preliminary study on the relationship between stimulated saliva and periodontal conditions in community-dwelling elderly people. J. Dent. 34, 692–698. 10.1016/j.jdent.2006.01.001 16473454

[B64] Holtfreter B. Schwahn C. Biffar R. Kocher T. (2009). Epidemiology of periodontal diseases in the study of health in pomerania. J. Clin. Periodontol. 36, 114–123. 10.1111/j.1600-051X.2008.01361.x 19207886

[B65] Hong J. S. Tian J. (2014). Prevalence of anxiety and depression and their risk factors in Chinese cancer patients. Support Care Cancer 22, 453–459. 10.1007/s00520-013-1997-y 24091720

[B66] Howren M. B. Lamkin D. M. Suls J. (2009). Associations of depression with C-reactive protein., IL-1., and IL-6: a meta-analysis. Psychosom. Med. 71, 171–186. 10.1097/PSY.0b013e3181907c1b 19188531

[B67] Jacka F. N. Berk M. (2012). Depression, diet and exercise. Med. J. Aust. 1, 21–23. 10.5694/mjao12.10508 25370279

[B68] Jani B. D. McLean G. Nicholl B. I. Barry S. J. Sattar N. Mair F. S.. (2015). Risk assessment and predicting outcomes in patients with depressive symptoms: a review of potential role of peripheral blood based biomarkers. Front. Hum. Neurosci. 9:18. 10.3389/fnhum.2015.00018 25698954 PMC4313702

[B69] Javed F. Warnakulasuriya S. (2016). Is there a relationship between periodontal disease and oral cancer? A systematic review of currently available evidence. Crit. Rev. Oncol. Hematol. 97, 197–205. 10.1016/j.critrevonc.2015.08.018 26343577

[B70] Johannsen A. Rylander G. Söder B. Asberg M. (2006). Dental plaque, gingival inflammation, and elevated levels of interleukin-6 and cortisol in gingival crevicular fluid from women with stress-related depression and exhaustion. J. Periodontol. 77, 1403–1409. 10.1902/jop.2006.050411 16937592

[B71] Johannsen A. Rydmark I. Söder B. Asberg M. (2007). Gingival inflammation, increased periodontal pocket depth and elevated interleukin-6 in gingival crevicular fluid of depressed women on long-term sick leave. J. Periodontal Res. 42, 546–552. 10.1111/j.1600-0765.2007.00980.x 17956468

[B72] Kandelman D. Petersen P. E. Ueda H. (2008). Oral health, general health, and quality of life in older people. Spec. Care Dentist 28, 224–236. 10.1111/j.1754-4505.2008.00045.x 19068063

[B73] Kang H. J. Kim S. Y. Bae K. Y. Kim S. W. Shin I. S. Yoon J. S.. (2015). Comorbidity of depression with physical disorders: research and clinical implications. Chonnam. Med. J. 51, 8–18. 10.4068/cmj.2015.51.1.8 25914875 PMC4406996

[B74] Kao C. F. Fang Y. S. Zhao Z. Kuo P. H. (2011). Prioritization and evaluation of depression candidate genes by combining multidimensional data resources. PLoS ONE 6:e18696. 10.1371/journal.pone.0018696 21494644 PMC3071871

[B75] Kassebaum N. J. Bernabé E. Dahiya M. Bhandari B. Murray C. J. Marcenes W. (2014a). Global burden of severe periodontitis in 1990-2010: a systematic review and meta-regression. J. Dent. Res. 93, 1045–1053. 10.1177/0022034514552491 25261053 PMC4293771

[B76] Kassebaum N. J. Bernabé E. Dahiya M. Bhandari B. Murray C. J. Marcenes W. (2014b). Global burden of severe tooth loss: a systematic review and meta-analysis. J. Dent. Res. 93, 20S–28S. 10.1177/0022034514537828 24947899 PMC4293725

[B77] Kessler R. C. Bromet E. J. (2013). The epidemiology of depression across cultures. Annu. Rev. Public Health. 3, 119–138. 10.1146/annurev-publhealth-031912-114409 23514317 PMC4100461

[B78] Khosravi R. Ka K. Huang T. Khalili S. Nguyen B. H. Nicolau B.. (2013). Tumor necrosis factor- α and interleukin-6: potential interorgan inflammatory mediators contributing to destructive periodontal disease in obesity or metabolic syndrome. Mediators Inflamm. 2013:728987. 10.1155/2013/728987 24068858 PMC3771422

[B79] Kinane D. Bouchard P. on behalf of Group E of the European Workshop on Periodontology (2008). Periodontal diseases and health: consensus report of the sixth European workshop on periodontology. J. Clin. Periodontol. 35, 333–337. 10.1111/j.1600-051x.2008.01278.x 18724860

[B80] Klages U. Weber A. G. Wehrbein H. (2005). Approximal plaque and gingival sulcus bleeding in routine dental care patients: relations to life stress, somatization and depression. J. Clin. Periodontol. 32, 575–582. 10.1111/j.1600-051X.2005.00716.x 15882214

[B81] Klein D. N. Kotov R. Bufferd S. J. (2011). Personality and depression: explanatory models and review of the evidence. Annu. Rev. Clin. Psychol. 7, 269–295. 10.1146/annurev-clinpsy-032210-104540 21166535 PMC3518491

[B82] Klimkiewicz A. Klimkiewicz J. Jakubczyk A. Kieres-Salomoñski I. Wojnar M. (2015). [Comorbidity of alcohol dependence with other psychiatric disorders. Part I. Epidemiology of dual diagnosis]. Psychiatr. Pol. 49, 265–275. 10.12740/PP/25704 26093591

[B83] Kocher T. Schwahn C. Gesch D. Bernhardt O. John U. Meisel P.. (2005). Risk determinants of periodontal disease–an analysis of the Study of Health in Pomerania (SHIP 0). J. Clin. Periodontol. 32, 59–67. 10.1111/j.1600-051X.2004.00629.x 15642060

[B84] Kronfeld-Schor N. Einat H. (2012). Circadian rhythms and depression: human psychopathology and animal models. Neuropharmacology 62, 101–114. 10.1016/j.neuropharm.2011.08.020 21871466

[B85] Kurer J. R. Watts T. L. Weinman J. Gower D. B. (1995). Psychological mood of regular dental attenders in relation to oral hygiene behaviour and gingival health. J. Clin. Periodontol. 22, 52–55. 10.1111/j.1600-051X.1995.tb01770.x 7706539

[B86] Kurosawa N. Shimizu K. Seki K. (2015). The development of depression-like behavior is consolidated by IL-6-induced activation of locus coeruleus neurons and IL-1β-induced elevated leptin levels in mice. Psychopharmacology (Berl). [Epub ahead of print]. 10.1007/s00213-015-4084-x 26385227

[B87] Leech M. T. Bartold P. M. (2015). The association between rheumatoid arthritis and periodontitis. Best Pract. Res. Clin. Rheumatol. 29, 189–201. 10.1016/j.berh.2015.03.001 26362738

[B88] Leonard B. Maes M. (2012). Mechanistic explanations how cell-mediated immune activation., inflammation and oxidative and nitrosative stress pathways and their sequels and concomitants play a role in the pathophysiology of unipolar depression. Neurosci. Biobehav. Rev. 36, 764–785. 10.1016/j.neubiorev.2011.12.005 22197082

[B89] Li Q. Xu C. Wu Y. Guo W. Zhang L. Liu Y.. (2011). [Relationship between the chronic periodontitis and the depression anxiety psychological factor]. Zhong Nan Da Xue Xue Bao Yi Xue Ban 36, 88–92. 10.3969/j.issn.1672-7347.2011.01.015 21311146

[B90] López R. Ramírez V. Marró P. Baelum V. (2012). Psychosocial distress and periodontitis in adolescents. Oral Health Prev. Dent. 10, 211–218. 10.3290/j.ohpd.a28516 23094263

[B91] Luca A. Luca M. Calandra C. (2013). Sleep disorders and depression: brief review of the literature, case report, and nonpharmacologic interventions for depression. Clin. Interv. Aging 8, 1033–1039. 10.2147/CIA.S47230 24019746 PMC3760296

[B92] Luger T. M. Suls J. Vander Weg M. W. (2014). How robust is the association between smoking and depression in adults? A meta-analysis using linear mixed-effects models. Addict. Behav. 39, 1418–1429. 10.1016/j.addbeh.2014.05.011 24935795

[B93] Luo J. Wu B. Zhao Q. Guo Q. Meng H. Yu L.. (2015). Association between tooth loss and cognitive function among 3063 Chinese older adults: a community-based study. PLoS ONE 10:e0120986. 10.1371/journal.pone.0120986 25803052 PMC4372206

[B94] Macedo C. R. Macedo E. C. Torloni M. R. Silva A. B. Prado G. F. (2014). Pharmacotherapy for sleep bruxism. Cochrane Database Syst. Rev. 10, CD005578. 10.1002/14651858.cd005578.pub2 25338726 PMC11033873

[B95] Maes M. (2011). Depression is an inflammatory disease, but cell-mediated immune activation is the key component of depression. Prog. Neuropsychopharmacol. Biol. Psychiatry 35, 664–675. 10.1016/j.pnpbp.2010.06.014 20599581

[B96] Maes M. Galecki P. Chang Y. S. Berk M. (2011b). A review on the oxidative and nitrosative stress (O&NS) pathways in major depression and their possible contribution to the (neuro)degenerative processes in that illness. Prog. Neuropsychopharmacol. Biol. Psychiatry 35, 676–692. 10.1016/j.pnpbp.2010.05.004 20471444

[B97] Maes M. Kubera M. Obuchowiczwa E. Goehler L. Brzeszcz J. (2011a). Depression's multiple comorbidities explained by (neuro)inflammatory and oxidative & nitrosative stress pathways. Neuro. Endocrinol. Lett. 32, 7–24. 21407167

[B98] Manosso L. M. Neis V. B. Moretti M. Daufenbach J. F. Freitas A. E. Colla A. R.. (2013). Antidepressant-like effect of α-tocopherol in a mouse model of depressive-like behavior induced by TNF-α. Prog. Neuropsychopharmacol. Biol. Psychiatry 46, 48–57. 10.1016/j.pnpbp.2013.06.012 23816813

[B99] Marques-Vidal P. Milagre V. (2006). Are oral health status and care associated with anxiety and depression? A study of Portuguese health science students. J. Public Health Dent. 66, 64–66. 10.1111/j.1752-7325.2006.tb02553.x 16570753

[B100] Márton K. Madléna M. Bánóczy J. Varga G. Fejérdy P. Sreebny L. M.. (2008). Unstimulated whole saliva flow rate in relation to sicca symptoms in Hungary. Oral. Dis. 14, 472–477. 10.1111/j.1601-0825.2007.01404.x 18938274

[B101] Mathers C. D. Loncar D. (2006). Projections of global mortality and burden of disease from 2002 to 2030. PLoS Med. 3:e442. 10.1371/journal.pmed.0030442 17132052 PMC1664601

[B102] Matthews J. C. You Z. Wadley V. G. Cushman M. Howard G. (2011). The association between self-reported tooth loss and cognitive function in the REasons for Geographic And Racial Differences in Stroke study: an assessment of potential pathways. J. Am. Dent. Assoc. 142, 379–390. 10.14219/jada.archive.2011.0192 21454843 PMC3744362

[B103] Mello B. S. Monte A. S. McIntyre R. S. Soczynska J. K. Custódio C. S. Cordeiro R. C.. (2013). Effects of doxycycline on depressive-like behavior in mice after lipopolysaccharide (LPS) administration. J. Psychiatr. Res. 47, 1521–1529. 10.1016/j.jpsychires.2013.06.008 23835040

[B104] Mendes D. C. Silva T. F. Barros Lde O. de Oliveira M. V. Vieira L. T. Haikal D. S.. (2013). Analysis of the normative conditions of oral health, depression and serotonin-transporter-linked promoter region polymorphisms in an elderly population. Geriatr. Gerontol. Int. 13, 98–106. 10.1111/j.1447-0594.2012.00867.x 22672136

[B105] Miller A. H. Maletic V. Raison C. L. (2009). Inflammation and its discontents: the role of cytokines in the pathophysiology of major depression. Biol. Psychiatry 65, 732–741. 10.1016/j.biopsych.2008.11.029 19150053 PMC2680424

[B106] Monteiro da Silva A. M. Oakley D. A. Newman H. N. Nohl F. S. Lloyd H. M. (1996). Psychosocial factors and adult onset rapidly progressive periodontitis. J. Clin. Periodontol. 23, 789–794. 10.1111/j.1600-051X.1996.tb00611.x 8877667

[B107] Morita M. Wang H. L. (2001). Association between oral malodor and adult periodontitis: review. J. Clin. Periodontol. 28, 813–819. 10.1034/j.1600-051x.2001.028009813.x 11493349

[B108] Moss M. E. Beck J. D. Kaplan B. H. Offenbacher S. Weintraub J. A. Koch G. G.. (1996). Exploratory case-control analysis of psychosocial factors and adult periodontitis. J. Periodontol. 67, 1060–1069. 10.1902/jop.1996.67.10s.1060 8910824

[B109] Moylan S. Berk M. Dean O. M. Samuni Y. Williams L. J. O'Neil A.. (2014). Oxidative & nitrosative stress in depression: why so much stress? Neurosci. Biobehav. Rev. 45, 46–62. 10.1016/j.neubiorev.2014.05.007 24858007

[B110] Moynihan P. Petersen P. E. (2004). Diet, nutrition and the prevention of dental diseases. Public Health Nutr. 7, 201–226. 10.1079/PHN2003589 14972061

[B111] Nagpal R. Yamashiro Y. Izumi Y. (2015). The two-way association of periodontal infection with systemic disorders: an overview. Mediators Inflamm. 2015:793898. 10.1155/2015/793898 26339142 PMC4539125

[B112] Nakada T. Kato T. Numabe Y. (2015). Effects of fatigue from sleep deprivation on experimental periodontitis in rats. J. Periodontal Res. 50, 131–137. 10.1111/jre.12189 24815330

[B113] Nishida M. Grossi S. G. Dunford R. G. Ho A. W. Trevisan M. Genco R. J. (2000). Dietary Vitamin C and the risk for periodontal disease. J. Periodontol. 71, 1215–1223. 10.1902/jop.2000.71.8.1215 10972636

[B114] Okoro C. A. Strine T. W. Eke P. I. Dhingra S. S. Balluz L. S. (2012). The association between depression and anxiety and use of oral health services and tooth loss. Community Dent. Oral Epidemiol. 40, 134–144. 10.1111/j.1600-0528.2011.00637.x 21883356

[B115] Paperwalla K. N. Levin T. T. Weiner J. Saravay S. M. (2004). Smoking and depression. Med. Clin. North Am. 88, 1483–94. 10.1016/j.mcna.2004.06.007 15464109

[B116] Pasco J. A. Nicholson G. C. Williams L. J. Jacka F. N. Henry M. J. Kotowicz M. A.. (2010). Association of high-sensitivity C-reactive protein with de novo major depression. Br. J. Psychiatry 197, 372–377. 10.1192/bjp.bp.109.076430 21037214

[B117] Patten S. B. (2013). Major depression epidemiology from a diathesis-stress conceptualization. BMC Psychiatry 13:19. 10.1186/1471-244X-13-19 23305517 PMC3549292

[B118] Payne J. B. Golub L. M. Thiele G. M. Mikuls T. R. (2015). The link between periodontitis and rheumatoid arthritis: a periodontist's perspective. Curr. Oral Health Rep. 2, 20–29. 10.1007/s40496-014-0040-9 25657894 PMC4312393

[B119] Peltzer K. Pengpid S. (2014). Oral health behaviour and social and health factors in university students from 26 low, middle and high income countries. Int. J. Environ. Res. Public Health 11, 12247–12260. 10.3390/ijerph111212247 25431876 PMC4276612

[B120] Persson G. R. Persson R. E. MacEntee C. I. Wyatt C. C. Hollender L. G. Kiyak H. A. (2003). Periodontitis and perceived risk for periodontitis in elders with evidence of depression. J. Clin. Periodontol. 30, 691–696. 10.1034/j.1600-051X.2003.00360.x 12887337

[B121] Peruzzo D. C. Benatti B. B. Ambrosano G. M. Nogueira-Filho G. R. Sallum E. A. Casati M. Z.. (2007). A systematic review of stress and psychological factors as possible risk factors for periodontal disease. J. Periodontol. 78, 1491–1504. 10.1902/jop.2007.060371 17668968

[B122] Petersen P. E. (2006). Oral health – general health interrelationships: health policy implications. Inside Dent. 1, 1–5.

[B123] Petersen P. E. Ogawa H. (2012). The global burden of periodontal disease: towards integration with chronic disease prevention and control. Periodontol. 2000 60, 15–39. 10.1111/j.1600-0757.2011.00425.x 22909104

[B124] Pham T. A. Ueno M. Shinada K. Kawaguchi Y. (2012). Factors affecting oral malodor in periodontitis and gingivitis patients. J. Investig. Clin. Dent. 3, 284–290. 10.1111/j.2041-1626.2012.00155.x 23129143

[B125] Pihlstrom B. L. Michalowicz B. S. Johnson N. W. (2005). Periodontal diseases. Lancet 366, 1809–1820. 10.1016/S0140-6736(05)67728-8 16298220

[B126] Pitiphat W. Merchant A. T. Rimm E. B. Joshipura K. J. (2003). Alcohol consumption increases periodontitis risk. J. Dent. Res. 82, 509–513. 10.1177/154405910308200704 12821709

[B127] Pratt L. A. Brody D. J. (2014). Depression and obesity in the U.S. adult household population., 2005–2010. NCHS Data Brief 167, 1–8. 25321386

[B128] Priyadarshini D. Nadig P. Deshpande N. Deshpande A. (2014). Role of psychotherapy in managing a case of generalised aggressive periodontitis. BMJ Case Rep. 2014:bcr2013200851. 10.1136/bcr-2013-200851 25035440 PMC4112343

[B129] Pussinen P. J. Paju S. Mäntylä P. Sorsa T. (2007). Serum microbial- and host-derived kers of periodontal diseases: a review. Curr. Med. Chem. 14, 2402–2412. 10.2174/092986707781745604 17896988

[B130] Ritchie C. S. (2007). Obesity and periodontal disease. Periodontol. 2000 44, 154–163. 10.1111/j.1600-0757.2007.00207. 17474931

[B131] Ronderos M. Ryder M. I. (2004). Risk assessment in clinical practice. Periodontol. 2000 34, 120–135. 10.1046/j.0906-6713.2003.003428.x 14717859

[B132] Roohafza H. Afghari P. Keshteli A. H. Vali A. Shirani M. Adibi P.. (2015). The relationship between tooth loss and psychological factors. Community Dent. Health 32, 16–19. 10.1922/CDH_3396Afshar04 26263587

[B133] Rosania A. E. Low K. G. McCormick C. M. Rosania D. A. (2009). Stress, depression, cortisol, and periodontal disease. J. Periodontol. 80, 260–266. 10.1902/jop.2009.080334 19186966

[B134] Rosenberger P. H. Jokl P. Ickovics J. (2006). Psychosocial factors and surgical outcomes: an evidence-based literature review. J. Am. Acad. Orthop. Surg. 14, 397–405. 10.1007/s12160-008-9078-z 16822887

[B135] Rosenquist J. N. Fowler J. H. Christakis N. A. (2011). Social network determinants of depression. Mol. Psychiatry 16, 273–281. 10.1038/mp.2010.13 20231839 PMC3832791

[B136] Rossetti C. Halfon O. Boutrel B. (2014). Controversies about a common etiology for eating and mood disorders. Front. Psychol. 5:1205. 10.3389/fpsyg.2014.01205 25386150 PMC4209809

[B137] Roy M. Tapadia M. G. Joshi S. Koch B. (2014). Molecular and genetic basis of depression. J. Genet. 93, 879–892. 10.1007/s12041-014-0449-x 25572252

[B138] Saintrain M. V. de Souza E. H. (2012). Impact of tooth loss on the quality of life. Gerodontology 29, e632–e636. 10.1111/j.1741-2358.2011.00535.x 21883422

[B139] Saletu A. Pirker-Frühauf H. Saletu F. Linzmayer L. Anderer P. Matejka M. (2005). Controlled clinical and psychometric studies on the relation between periodontitis and depressive mood. J. Clin. Periodontol. 32, 1219–1225. 10.1111/j.1600-051X.2005.00855.x 16268998

[B140] Samnieng P. Ueno M. Shinada K. Zaitsu T. Wright F. A. Kawaguchi Y. (2012). Association of hyposalivation with oral function, nutrition and oral health in community-dwelling elderly Thai. Community Dent. Health 29, 117–123. 10.1922/CDH_2690Ueno07 22482262

[B141] Sarkar S. Schaefer M. (2014). Antidepressant pretreatment for the prevention of interferon alfa-associated depression: a systematic review and meta-analysis. Psychosomatics 55, 221–234. 10.1016/j.psym.2013.06.015 24012293

[B142] Semenkovich K. Brown M. E. Svrakic D. M. Lustman P. J. (2015). Depression in type 2 diabetes mellitus: prevalence, impact, and treatment. Drugs 75, 577–587. 10.1007/s40265-015-0347-4 25851098

[B143] Serafini G. Montebovi F. Lamis D. A. Erbuto D. Girardi P. Amore M.. (2015). Associations among depression, suicidal behavior, and quality of life in patients with human immunodeficiency virus. World J. Virol. 4, 303–312. 10.5501/wjv.v4.i3.303 26279991 PMC4534821

[B144] Sheiham A. Watt R. G. (2000). The common risk factor approach: a rational basis for promoting oral health. Community Dent. Oral Epidemiol. 28, 399–406. 10.1034/j.1600-0528.2000.028006399.x 11106011

[B145] Silveira E. M. Piccinin F. B. Gomes S. C. Oppermann R. V. Rösing C. K. (2012). Effect of gingivitis treatment on the breath of chronic periodontitis patients. Oral Health Prev. Dent. 10, 93–100. 10.3290/j.ohpd.a25703 22908093

[B146] Slavich G. M. Irwin M. R. (2014). From stress to inflammation and major depressive disorder: a social signal transduction theory of depression. Psychol. Bull. 140, 774–815. 10.1037/a0035302 24417575 PMC4006295

[B147] Smith D. J. Court H. McLean G. Martin D. Langan M. J. Guthrie B.. (2014). Depression and multimorbidity: a cross-sectional study of 1,751,841 patients in primary care. J. Clin. Psychiatry 75, 1202–1208. 10.4088/jcp.14m09147 25470083

[B148] Sobel R. M. Lotkowski S. Mandel S. (2005). Update on depression in neurologic illness: stroke, epilepsy, and multiple sclerosis. Curr. Psychiatry Rep. 7, 396–403. 10.1007/s11920-005-0043-2 16216161

[B149] Soletti A. C. Gaio E. J. Rosing C. K. (2009). Effect of neonatal clomipramine in the pathogenesis of ligature-induced periodontitis in Lewis rats. Acta Odontol. Scand. 67, 94–98. 10.1080/00016350802683822 19169913

[B150] Solis A. C. Lotufo R. F. Pannuti C. M. Brunheiro E. C. Marques A. H. Lotufo-Neto F. (2004). Association of periodontal disease to anxiety and depression symptoms, and psychosocial stress factors. J. Clin. Periodontol. 31, 633–638. 10.1111/j.1600-051X.2004.00538.x 15257740

[B151] Solis A. C. Marques A. H. Pannuti C. M. Lotufo R. F. Lotufo-Neto F. (2014). Evaluation of periodontitis in hospital outpatients with major depressive disorder. J. Periodontal Res. 49, 77–84. 10.1111/jre.12082 23586804 PMC4479258

[B152] Syrjälä A. M. Raatikainen L. Komulainen K. Knuuttila M. Ruoppi P. Hartikainen S.. (2011). Salivary flow rate and periodontal infection - a study among subjects aged 75 years or older. Oral Dis. 17, 387–392. 10.1111/j.1601-0825.2010.01764.x 21114589

[B153] Tezal M. Grossi S. G. Ho A. W. Genco R. J. (2004). Alcohol consumption and periodontal disease. The Third National Health and Nutrition Examination Survey. J. Clin. Periodontol. 31, 484–488. 10.1111/j.1600-051X.2004.00503.x 15191580

[B154] Thomson W. M. Poulton R. Broadbent J. M. Al-Kubaisy S. (2006). Xerostomia and medications among 32-year-olds. Acta Odontol. Scand. 64, 249–254. 10.1080/00016350600633243 16829502 PMC2249168

[B155] Thomson W. M. Sheiham A. Spencer A. J. (2012). Sociobehavioral aspects of periodontal disease. Periodontol. 2000 60, 54–63. 10.1111/j.1600-0757.2011.00405.x 22909106

[B156] Tsai C. C. Chou H. H. Wu T. L. Yang Y. H. Ho K. Y. Wu Y. M.. (2008). The levels of volatile sulfur compounds in mouth air from patients with chronic periodontitis. J. Periodontal Res. 43, 186–193. 10.1111/j.1600-0765.2007.01011.x 18302621

[B157] Turek F. W. (2007). From circadian rhythms to clock genes in depression. Int. Clin. Psychopharmacol. 22, S1–S8. 10.1097/01.yic.0000277956.93777.6a 17917561

[B158] Urzua I. Mendoza C. Arteaga O. Rodríguez G. Cabello R. Faleiros S.. (2012). Dental caries prevalence and tooth loss in chilean adult population: first national dental examination survey. Int. J. Dent. 2012:810170. 10.1155/2012/810170 23316234 PMC3536045

[B159] Ustün T. B. Ayuso-Mateos J. L. Chatterji S. Mathers C. Murray C. J. (2004). Global burden of depressive disorders in the year 2000. Br. J. Psychiatry 184, 386–392. 10.1192/bjp.184.5.386 15123501

[B160] Valkanova V. Ebmeier K. P. Allan C. L. (2013). CRP., IL-6 and depression: a systematic review and meta-analysis of longitudinal studies. J. Affect. Disord. 150, 736–744. 10.1016/j.jad.2013.06.004 23870425

[B161] Warren K. R. Postolache T. T. Groer M. E. Pinjari O. Kelly D. L. Reynolds M. A. (2014). Role of chronic stress and depression in periodontal diseases. Periodontol. 2000 64, 127–138. 10.1111/prd.12036 24320960 PMC7167640

[B162] Watt R. G. (2007). From victim blaming to upstream action: tackling the social determinants of oral health inequalities. Community Dent. Oral Epidemiol. 35, 1–11. 10.1111/j.1600-0528.2007.00348.x 17244132

[B163] Watt R. G. Petersen P. E. (2012). Periodontal health through public health - the case for oral health promotion. Periodontol. 2000 60, 147–155. 10.1111/j.1600-0757.2011.00426.x 22909112

[B164] Watt R. G. Sheiham A. (2012). Integrating the common risk factor approach into a social determinants framework. Community. Dent. Oral. Epidemiol. 40, 289–296. 10.1111/j.1600-0528.2012.00680.x 22429083

[B165] Williams R. C. Offenbacher S. (2000). Periodontal medicine: the emergence of a new branch of periodontology. Periodontol. 2000 23, 9–12. 10.1034/j.1600-0757.2000.2230101.x 11276770

[B166] Wu S. Barugh A. Macleod M. Mead G. (2014). Psychological associations of poststroke fatigue: a systematic review and meta-analysis. Stroke 45, 1778–1783. 10.1161/STROKEAHA.113.004584 24781083 PMC4928705

[B167] Yamamoto T. Kondo K. Misawa J. Hirai H. Nakade M. Aida J.. (2012). Dental status and incident falls among older Japanese: a prospective cohort study. BMJ Open 2, e001262. 10.1136/bmjopen-2012-001262 22855628 PMC4400665

[B168] Yuen H. K. Hant F. N. Hatfield C. Summerlin L. M. Smith E. A. Silver R. M. (2014). Factors associated with oral hygiene practices among adults with systemic sclerosis. Int. J. Dent. Hyg. 12, 180–186. 10.1111/idh.12056 24128049 PMC3988272

[B169] Zhu L. Wei T. Gao J. Chang X. He H. Miao M.. (2015). Salidroside attenuates lipopolysaccharide (LPS) induced serum cytokines and depressive-like behavior in mice. Neurosci. Lett. 606, 1–6. 10.1016/j.neulet.2015.08.025 26300543

